# Better conversations: a language and communication intervention for aphasia in posterior cortical atrophy

**DOI:** 10.1080/13554794.2022.2125326

**Published:** 2022-09-21

**Authors:** A. Volkmer, C Farrington-Douglas, Sj Crutch, S Beeke, Jd Warren, Kxx Yong

**Affiliations:** a Department of Psychology and Language Sciences, University College London, London, UK; b Dementia Research Centre, University College London, London, UK

**Keywords:** Posterior cortical atrophy, speech and language therapy, conversation, communication partner training, primary progressive aphasia

## Abstract

Posterior cortical atrophy (PCA) describes a neurodegenerative syndrome characterized by progressive difficulties in cortical visual and other posterior cortical functions consistent with parieto-occipital and occipito-temporal involvement. It is increasingly recognized that many patients develop difficulties with other aspects of daily living, in particular, with language and communication. We present a case emphasizing how language difficulties may emerge in PCA. Difficulties are interpreted as arising from interacting effects of linguistic deficits and impaired detection of nonverbal (particularly, visual) turns that normally facilitate, schedule, and disambiguate the exchange of verbal messages between speakers. We propose that relatively simple speech and language therapy interventions may hold promise in addressing language and communication difficulties as secondary features of PCA by targeting the behaviors of both the person with PCA and their communication partners.

## Introduction

Posterior cortical atrophy (PCA) describes a neurodegenerative syndrome characterized by progressive difficulties in cortical visual and other posterior cortical functions. Posterior atrophy may be evident bilaterally, right or to a lesser degree left lateralized (Crutch et al., 2017; Ryan et al., 2014) PCA is most commonly underpinned by Alzheimer’s disease pathology and while uncommon (affecting perhaps 2.5/100,000 people aged between 30 and 64: Chiari et al., 2021), it has disproportionate importance as the paradigmatic ‘visual’ dementia. Although visual and visuomotor impairments such as space and object perception deficits, simultanagnosia, constructional dyspraxia and optic ataxia, have rightly been emphasized in the literature on PCA (Crutch et al., 2017), it is increasingly recognized that many patients develop difficulties with other aspects of daily living, in particular, with language and communication.

Communication difficulties relating to visual impairments, such as alexia, are frequent consequences of PCA (80–95%; McMonagle et al., 2006; Mendez et al., 2002; Yong et al., 2014; Yong et al., 2015) and agraphia are included as core cognitive features in PCA consensus criteria (Crutch et al., 2017). *Peripheral alexia in PCA includes particular difficulties perceiving single words in cursive, larger font with unfamiliar formats (Yong et al., 2014) as well as a characteristic tendency for people getting lost when moving within or between lines during text reading (Yong et al., 2015). The profile of peripheral alexia in PCA has been described as “apperceptive” (Mendez et al., 2002) or as “crowding dyslexia*” (Crutch & Warrington, 2009). However, early anomia and reduced verbal fluency has also been reported as relatively common in the research literature (Crutch et al., 2013; Schott & Crutch, 2019; Tang-Wai et al., 2004). PCA has also been shown to degrade the parsing of the auditory environment (Hardy et al., 2020) which could cause or exacerbate comprehension difficulties. The specific characteristics of the speech and language impairment in PCA have been described as not dissimilar, though milder, to those that people with logopenic variant of primary progressive aphasia lvPPA present with (Fitzpatrick et al., 2019). This likely reflects breakdown of a shared temporo-parietal neural network that has been described in PCA (Crutch et al., 2013; Fitzpatrick et al., 2019; Schott & Crutch, 2019; Tang-Wai et al., 2004) characterized by anomia, reduced phonological working memory and impaired syntactic comprehension (Crutch et al., 2013).

However, less well appreciated is the extent to which PCA may impact interaction and the natural conversational exchanges of daily life. Conversation is likely to be particularly vulnerable in PCA, due to the interacting effects of linguistic deficits and impaired detection of nonverbal (particularly, visual) signals that normally facilitate, schedule and disambiguate the exchange of verbal turn sequences between speakers. The combined effect of visual and communication difficulties on interaction may explain the impact of these communication difficulties in the everyday lives of people with PCA. We know from Conversation Analysis (CA) studies that people with lvPPA may increasingly rely on their partners to repair conversational breakdown, resulting in extended turn sequences (where the speaker self-selects to take the next turn and thus continues taking turns) (Taylor et al., 2014). The use of test-questions by a partner (whereby a communication partner asks a question despite knowing the answer), is common in conversations with people with dementia and often considered a behavior that risks exposing the limitations of a person with a communication difficulty, and consequently a lack of competence (Kindell et al., 2017; Wilkinson, 2015). These conversation difficulties ultimately lead to the common experience of loss of interactional competence, meaning the person feels that they do not have the ability to participate in social situations with other people (Kindell et al., 2017; Wilkinson, 2015).

The extent of language symptoms in PCA and their similarity to lvPPA have prompted proposals that speech and language interventions developed for lvPPA may be helpful for people with PCA (Crutch et al., 2013; Fitzpatrick et al., 2019). There is evidence that lexical word retrieval interventions (based on self-cueing, reading, repetition, and recall) have a positive effect, resulting in increased accuracy in retrieval of trained and untrained words for people living with lvPPA (Croot, 2018; Graff-Radford et al., 2021; Jokel et al., 2014); such interventions aim to maintain or restore language impairment through systematic practice of target vocabulary. Alongside frustration and loss of competence experienced by the person with PPA, communication partners (often a spouse, partner or adult child) may experience sense of helplessness (Taylor et al., 2014). Correspondingly, communication partner training has been favored when working with people with PPA (Volkmer et al., 2019; Volkmer, 2020) in order to address the impact of the language impairment in conversation and support identification of practical strategies to enhance interactions between the person and their communication partner. However, despite the promise of lexical word retrieval and communication partner training interventions and evidence-base aids supporting diminished reading for people with PCA (Suarez-Gonzalez et al., 2019; Yong et al., 2015), there are essentially no studies of speech and language interventions for people with PCA.

One communication partner training intervention developed for people with PPA and their communication partners is Better Conversations with PPA (BCPPA; Volkmer et al., 2018, 2021). The development of the BCPPA intervention has been reported elsewhere (Volkmer et al., 2021) and the protocol for the intervention has been described using the TiDIER checklist (Volkmer et al., 2018).

The current investigation outlines an individual, MM, who exhibited prominent and disabling language and communication difficulties as secondary features of PCA, evident on patient cognitive and communication partner outcomes. Following referral to a speech and language therapy service, the Better Conversations with PPA intervention was administered to MM; here we document the implementation and effects of this intervention.

## Case report

A 60-year-old gentleman (MM) was referred to the clinical speech and language therapy service with a progressive aphasia and significant frustration due to difficulty conversing with his wife. Given the service was a research site for the Better Conversation with Primary Progressive Aphasia (BCPPA) study, MM was identified as eligible (for study protocol and inclusion criteria see Volkmer et al., 2018). MM and his partner GG were invited to, and consented to, participate in the study. Ethical approval for the study was granted by London-Camden and Kings Cross Research Ethics Committee (reference: 17/LO/0357) and an amendment to include remote delivery approved on 6 August 2020.

MM’s symptoms had initially presented some seven years previously, with insidious deterioration in visuospatial functioning, praxis and arithmetic skills, despite relatively spared language, memory and executive skills fulfilling PCA consensus criteria (Crutch et al., 2017). There has been an increase in patients with PCA being referred to the speech and language therapy clinic over the last 3-years. MM presented with both a typical PCA phenotype and also subsequently developed prominent language features which may have been exacerbated by visual disturbances. It was not within the clinical remit of the speech and language therapy service to assess MMs visuo-perceptual skills and there were no neuropsychological test scores available at this time. Incidentally however, MM had participated in a previous research study within the Dementia Research Center and neuropsychological test data was made available following his participation in the study. Subsequently and at the time of study participation, MM was taking Memantine (20 mg) and Donepezil (10 mg). Routine clinical speech and language assessment using the Comprehensive Aphasia Test (Swinburn et al., 2004; CAT) was completed with MM to ascertain an overview of his areas of communication strength and difficulty. The CAT is routinely used to assess people with PPA across clinical speech and language therapy services in the UK (Volkmer et al., 2019). At the time of referral to speech and language therapy, MM exhibited mild linguistic deficits affecting naming, syntactic comprehension and non-word repetition (see Table 1 for an overview of MM’s neuropsychological and language testing). This profile was consistent with Gorno-Tempini et al. (2011) criteria for lvPPA outside exclusionary criteria (initial visuospatial impairment owing to PCA).

**Table 1.** Summary of MM’s performance on general neuropsychological and neurolinguistic tests.

General neuropsychology	Raw scores	Norms/comment
MMSE^1^	22/30	
Short Recognition Memory Test for words^2^*	25/25	>50^th^ %ile
(joint auditory/visual presentation)		
Concrete Synonyms test^3^	23/25	>50^th^ %ile
Naming (verbal description)	19/20	>50^th^ %ile
Cognitive estimates^4^ (error score)	0	>50^th^ %ile
Calculation (GDA^5^)*	1/24	<1st %ile
Spelling (GDST^6^- Set B, first 20 items)*	16/20	10-25^th^%ile
Gesture production test^7^	15/15	-
Digit span (forwards)	12/12	>50^th^ %ile
Digit span (backwards)	5/12	25-50^th^ %ile
*Early visual processing*		
Visual acuity (CORVIST^8^): Snellen	6/9	Normal
Figure-ground discrimination (VOSP^9^)	20/20	>50^th^ %ile
Shape discrimination – Efron squares^10^	19/20	Healthy control do not make errors
Hue discrimination (CORVIST)	3/4	Impaired
*Visuoperceptual processing*		
Fragmented letters (VOSP)*	12/20	<5^th^ %ile
Object Decision (VOSP)*	13/20	CLA: <5th%ile; FOL: 10^th^-25^th^%ile
Unusual and usual views^11^: Unusual	11/20	<1st %ile
Unusual and usual views^11^: Usual	19/20	Within normal range
*Visuospatial processing*		
Number location (VOSP)*	2/10	<5^th^ %ile
Dot counting (VOSP)*	4/10	<5^th^ %ile
A Cancellation^12^: Completion time	56	<5th %ile
A Cancellation^12^: Number of letters missed	0	-
CORVIST reading test	16/16	-
**Language assessment using CAT13**		
*Receptive language*		
Spoken word picture matching (visual presentation)	26/30	Within normal range
Spoken sentence picture matching (visual presentation)	22/32	<5^th^ %ile
Comprehension of spoken paragraphs	4/4	Within normal range
Total spoken comprehension score	52/66	<5^th^ %ile
*Expressive language*		
Repetition of real words	32/32	Within normal range
Repetition of complex words	6/6	Within normal range
Repetition of non-words	6/10	<5^th^ %ile
Digit span (forwards)	14/14	Within normal range
Repetition of sentences	12/12	Within normal range
Naming objects (visual presentation)	46/48	Within normal range
Naming actions (visual presentation)	4/10	<5^th^ %ile
Picture description *(visual presentation)*	23	<5^th^ %ile

*Behavioral screening tests supportive of PCA diagnosis. ^1^ Mini-Mental State Examination (MMSE: Folstein et al., 1975). ^2^ Warrington (1996). ^3^ Warrington et al. (1998). ^4^ Shallice and Evans (1978). ^5^ Graded Difficulty Arithmetic test (GDA; Jackson & Warrington, 1986). ^6^ Graded Difficulty Spelling Test (GDST; Baxter & Warrington, 1994). ^7^ Crutch (unpublished). ^8^ Cortical Visual Screening Test (CORVIST; James et al., 2001).^9^ Visual Object and Space Perception Battery (VOSP; Warrington & James, 1991). ^10^ Efron (1968). ^11^ Warrington and James (1988). ^12^ Willison and Warrington (1992) ^13^ Comprehensive Aphasia Test (CAT; Swinburn et al., 2004).

MM’s difficulties on repetition of non-words, a test that requires the individual to repeat non-meaningful words, thus not allowing them to use their stored semantic or lexical knowledge, demonstrated problems at the phonological level. This points toward a phonological buffer impairment with similarities to that of an individual with logopenic PPA (Crutch et al., 2013).

At the time that MM participated in the study, during the COVID-19 pandemic, all clinical services were being delivered remotely, therefore this report describes speech and language assessments, outcome measures and intervention delivered using the video conferencing platform approved by the local NHS trust at that time (Attend Anywhere).

### Intervention procedures

As is standard in the BCPPA intervention, prior to commencing the therapy sessions MM and GG prepared a 10-minute video recording of themselves in a natural everyday conversation at home and shared this with the speech and language therapist. They were provided with a handout to support them to identify a possible topic, a coproduced resource available as part of the BCPPA intervention. To inform intervention delivery, this video was analyzed by the speech and language therapist using the Better Conversation Observation Checklist (Beeke et al., 2021). This involves the speech and language therapist watching the video recording, often repeatedly, and making notes about behaviors or ‘practices’ that are facilitators (that enhance the flow) and barriers (that result in conversation breakdown). This analysis revealed MM spoke significantly less than GG because he was unable to initiate his own conversational turns. When GG indicated by small gestures, facial expression and eye gaze, that she had completed her turn, MM would often not respond. Where an audible pause ensued, he was observed to attempt to fill this, initiating word searching behaviors such as averting eye gaze, or gesturing, until his wife retook the conversational floor. MM’s effective exclusion from the conversation was compounded by his GG’s distraction by intercurrent activities (looking at her phone and care tasks that require visual attention such as preparing food, etc whilst engaged in the interaction), which meant that she was frequently unaware of his attempts to communicate.

MM and his wife received four 1-hour sessions of person-centered speech and language therapy using the Better Conversations with Primary Progressive Aphasia, BCPPA, protocol (A full description of the intervention has been published in Volkmer et al., 2018; Volkmer, 2020). BCPPA was co-produced with people with PPA, their communication partners and speech and language therapists using the Medical Research Council guidance on developing complex interventions and is informed by applied conversation analysis and behavior change theory (Volkmer et al., 2021). Briefly, the intervention comprised four 1-hour interventions sessions delivered weekly by a trained speech and language therapist (SLT). Table 2 provides an overview of the aims and tasks of the four BCPPA intervention sessions. During the first session, the SLT provided an overview of “How conversation works” and “What can go wrong in conversation” supported by accessible aphasia-friendly handouts. The SLT then presented MM and GG with three carefully selected 30-second video clips. These clips were selected to reflect examples of behaviors than seemed to be obvious barriers (such as GG becoming distracted and MM not being able to see cues to support turn taking) or obvious facilitators (such as GG taking time to observe MM’s attempts to initiate a turn), The therapist then facilitated a discussion supporting the dyad to analyze and reflect on the recordings. This underpinned the identification of goals (using Goal Attainment Scaling; Turner-Stokes, 2009) to work on in therapy, identifying what behaviors MM and GG wished to do more or less of, before embarking on a period of practice, problem solving and further practice. MM and GG identified two specific goals, during session 2, with the overarching aim of allowing MM more opportunities for turn taking in conversation. The first goal was for GG to slow down to give MM time to contribute and the second goal was to set protected times for conversation each day for a relaxed, positive connection. Consequently, the remaining sessions (3 & 4) focused on practicing and refining the target behaviors identified in their goals.

**Table 2.** BCPPA session aims and tasks.

Session	Aims	Tasks
1. What is Conversation?	Discuss aims of therapy Discuss and explore what conversation is and how it can go wrong Initial viewing of their own video	Give an overview of therapy Explanation of how conversation works Initial discussions and exploration of how PPA affects conversations Initial viewing of a video Home based task in preparation for next session
2. Goal Setting	Identify barriers and facilitators in their own conversation Set goals for therapy based on this discussion	View video of their own conversation Identify areas of strength and areas where there are problems in the conversation Identifying goals to target in therapy Home based task in preparation for next session
3. Practice	Practice conversation using the strategies identified during goal setting Problem solve any issues that have arisen in using identified strategies in conversations outside of therapy sessions	Role play or record the couple in the session practicing strategy use Identify when and where they will use these strategies at home and if not why not. Home based task in preparation for next session
4. Problem solving and planning for the future	Consider planning for future changes in communication 2. Practice conversation using the strategies identified during goal setting	Role play or record the couple in the session practicing strategy use Review goals set in session 2 Discuss and plan for future changes in communication

NB: Each session was accompanied by relevant handouts and homebased tasks which have been published on the UCL eXtend website for collaborators in the BCPPA pilot study and are anticipated to be publicly available following a future effectiveness study.

### Outcomes

The primary aim of BCPPA is to support identification of practical strategies to enhance interactions between the person and their communication partner, thereby increasing confidence and reducing frustration in conversation. Correspondingly, pre and post intervention outcome data was collected from MM, by the therapist in a session, using two self-rating questionnaires. The Aphasia Impact Questionnaire (Swinburn et al., 2015) is a tool designed to measure the impact of living with aphasia across three domains of communication, participation, and emotional well-being. The participant rates their response to 19 questions on a scale from 0 to 4 defined pictorially in an accessible format. Lower scores represent a better outcome, with additional definitions of 0 and 4 provided depending on the context of the question (for example, when asked “How easy was it to talk to someone close to you?” 0 is defined as “no problem,” whilst 4 is defined as “impossible”). The Communication Confidence Rating Scale for Aphasia (Babbitt et al., 2011) was also used. This is a ten-item questionnaire of communication confidence that uses a self-rating scale from 0 to 10 designed for people with aphasia. Higher scores represent a better outcome, but there are no definitions or qualifiers beyond this. Pre-to-post intervention scores on the Aphasia Impact Questionnaire were compared using a McNemar Test (SPSS version 27). McNemar has been recommended for use to examine change in a single subject using self-report questionnaires (Caronni & Sciumè, 2017).

MM and GG’s goals were recorded using Goal Attainment Scaling (GAS; Turner-Stokes, 2009). They were asked to rate the importance of each goal (on a scale from 1 to 3, with 1 = more important and 3 = least important) and whether they felt the goal was achievable or not. Following the intervention, MM and GG were invited to rate whether they had achieved their goals (+2 = overachieved in their opinion, +1 = somewhat overachieved, 0 = achieved, −1 = not achieved). The guide to using GAS provides guidance on calculating the baseline and attainment score (Turner-Stokes, 2009). In this study, all goals were assigned a baseline of −1 and both a baseline and attainment GAS score calculated using the above formula. A score of 50 is considered to reflect that overall goals have been achieved precisely, whilst a score of more than 60 indicating better than expected results.

Regular review and re-assessment using language testing is routine in clinical speech and language therapy to inform an understanding of progression of disease. Consequently, language testing, specifically, picture description and naming subtests from the CAT^13^, were also repeated.

## Results

At a routine clinical review (as is recommend for clinical management of progressive communication disorders; Volkmer et al., 2022), scheduled two months after completing the intervention, MM and GG reported ongoing benefit of the therapeutic strategies in their mutual communication. They rated both goals they had set as overachieved (see Table 2). GG reported having a set time to chat helped her to protect time to use her strategies, whilst their aptly named “bright ideas book,” where they decided to write down reflections on their conversations, highlighted the positive experiences, such as MM laughing and reporting feeling more relaxed. This book was initiated by the participants, during the practice phase of the intervention, in line with the individualized approach which is central to this intervention. The guide to using GAS provides guidance on calculating the baseline and attainment score (Turner-Stokes, 2009). MM and GG’s baseline score was 38 and the post-intervention attainment score was 62.

Four months following the baseline evaluation, and two months after therapy finished MM reported a modest reduction in the impact of the aphasia on his life on the Aphasia Impact Questionnaire, decreasing from a total score of 32/76 to 29/76. There was no significant difference in score pre to post intervention on the Aphasia Impact Questionnaire (McNemar–Bowker Test χ^2^ = 3.33, df = 1, p = 0.649). Eight item-level scores changed and 11 remained the same post intervention. Small changes (1 point) in scores were observed on 6 items (four indicated a worsening and two indicated an improvement), one item (How were things with friends?) improved from 4 (impossible), to 1 (with 0 representing no problem) and another item (this week have you felt isolated) improved from 4 to 2. There was no change in MMs total rating on the CCRSA. Scores on language testing were maintained, though an improvement in picture description scores was observed (from 23 pre-treatment to 41 post-treatment; see Table 3 for a summary of all scores).

**Table 3.** Pre- and post-intervention data collated from MM and GG.

	Max score	Pre intervention	Post intervention
Goal Attainment Scaling^14^	100 (higher indicates greater goal achievement)	38	62
Aphasia Impact Questionnaire^15^	0 (lower indicates reduction in impact)	32/76	29/76
Communication Confidence Rating Scale in Aphasia^16^	1000 (higher indicates increased confidence)	720/1000	720/1000
Naming of objects – subtest of CAT^13^	24	23/24	24/24
Picture description – subtest of CAT			
Appropriate Information Carrying Words		20	34
Inappropriate Information Carrying Words		6	**7**
Syntactic variety		2	6
Grammatical well-formedness		4	5
Speech		3	3
Total	Unbounded maximum score (higher indicates better language ability)	23 (t score = 55)	41 (t score = 63)

^14^Goal Attainment Scaling (GAS; Turner-Stokes, 2009); ^15^ The Aphasia Impact Questionnaire (Swinburn, 2013); ^16^ Communication Confidence Rating Scale for Aphasia (Babbitt et al., 2011); ^13^ Comprehensive Aphasia Test (CAT; Swinburn et al., 2004).

## Discussion

This case underlines the importance of nonverbal cues in the ostensibly ‘verbal’ communication we engage in every day. The typical flow of dialogue depends on an exquisitely timed conversational pas de deux that under normal circumstances is largely unconscious, but which can (as here) present therapeutic opportunities when explicitly discussed with people. MM’s example additionally illustrates how the combination of perceptual deficits and language difficulties in PCA may have far-reaching and unanticipated impacts on daily living. Of course, people who are congenitally blind are able to converse effectively without seeing their interlocutor, and many of us are capable of holding conversations on the telephone. The conversational difficulties MM experienced are likely to have reflected the interaction of progressive visual and primary linguistic impairment (especially, word finding difficulty). These were potentially further amplified by disordered speech perception: the cadential signals that punctuate dialogue are auditory as well as visual, and PCA has been shown to degrade the parsing of the auditory environment (Hardy et al., 2020). This case adds to previous CA studies in PPA, which have demonstrated extended turn sequences associated with collaborative repair of word errors (Taylor et al., 2014), MM’s case indicates that the impact of visual and language impairments may also result in extended turn sequences and potential missed turn taking opportunities. Though CA was not formally applied in this case example, further research employing these methods would be a valuable addition to the field.

Despite a logopenic type aphasia being very common across AD, perhaps especially atypical forms (Graff-Radford et al., 2021), the disparity between the severity of the language difficulties observed (relatively mild), and the significant impact it has for MM and his partner is striking. A recent scoping review identified only seven studies examining language impairments in PCA (Suarez-Gonzales et al., 2021), with no information found on the severity of these difficulties. We know that PPA can have a negative impact on quality of life for the person living with the diagnosis (Ruggero et al., 2019), it is therefore unsurprising that people with PCA and their partners experience similar negative consequences. It is perhaps due to the importance of communication as a tool to compensate for visual perceptual deficits, that communication becomes so important for people with PCA, and should signal a prompt referral to speech and language therapy

This case report also represents a valuable first study exploring speech and language interventions for people with PCA. In our experience, the increased awareness of the potential benefits of speech and language therapy for people with progressive language difficulties has contributed to an increase in referrals, including for conditions such as PCA where the primary difficulties are mostly considered outside (nonvisual) language domains. Given that MM and his wife’s conversations improved as per their self-evaluation of goal attainment, and he is less frustrated, this reaffirms that people with PCA can derive meaningful benefit from relatively simple interventions that compensate for their communication difficulties. Watching recordings has been identified as a key component in motivating behavior change in communication partner training, such as BCPPA (Johnson, 2015). In its current form, BCPPA includes handouts and video recordings of people’s own conversations, that, due to their visual perceptual impairments, may be difficult for people with PCA to access (Crutch et al., 2017). Despite this barrier, the multimodal nature of a video recording enabled MM and GG to see and hear the video recording and participate in discussion throughout the intervention. Future work in this area should investigate how the visuo-perceptual needs of people with PCA in combination with language difficulties could inform coproduction of a refined BCPPA intervention. A refined BCPPA might maximize accessibility for people with prominent cortical visual loss, with relevance not only to PCA but also related clinical phenotypes (e.g., lvPPA or corticobasal syndrome with prominent visuospatial deficits).

Goal review demonstrated that MM and GG both benefited from the intervention, and the self-rating measures indicated maintenance of confidence and modest improvement in quality of life scores (albeit non-significant). In progressive conditions, maintenance must be considered as much a worthy outcome as improvement (Croot et al., 2009). An important limitation of this study is the lack of objective measures of treatment effect. GAS goals are often used as a secondary outcome measure, with an objective measure as the primary outcome measure. A future study of this nature could use a method not dissimilar to that described by Best et al. (2016). They used a modified CA approach and masked raters to code and count behaviors observed in video recordings of everyday conversations before and after an intervention for a person with stroke aphasia and their partner. Gauging the partner’s perception of the patient’s language abilities pre- and post-treatment would also provide helpful data, though being aware of the risk of proxy-bias (Wu et al., 2020). Importantly, a minimal important difference, the smallest difference that patients perceive as beneficial and therefore clinically significant, is not always statistically significant (Caronni & Sciumè, 2017). Thus, measuring the impact of an intervention in a progressive disease is particularly complex and remains an area of some debate in the speech and language therapy field.

However, a limitation of all measures and assessments used in this case study are that they are not tailored to the needs of someone with PCA. Aside from the fact that the language assessment (the CAT subtests) are not standardized for people with progressive aphasia or PCA (Swinburn et al., 2004), it is notable that this tool depends largely on visually presented stimuli (including picture description, naming of object/verb pictures and selecting pictures from verbal stimuli). Any of MM’s scores on these subtests will be confounded by his visual perceptual impairment. Importantly, MM scored as impaired on repetition of non-words, a subtest that requires no visual processing, indicating the presence of a phonological processing deficit. Further research exploring the language profile of individuals with PCA, and the impact on discourse samples, conversation, and quality of life would benefit people living with the disease, their families, clinicians, and researchers. This, in turn, would also drive the much-needed research developing interventions, such as evidence-based reading aids mitigating peripheral dyslexia (Suarez-Gonzalez et al., 2019; Yong et al., 2015), and maximizing proposed benefits of psychological, occupational therapy, and peer-to-peer support through improved functional communication (Graff-Radford et al., 2021; Schott & Crutch, 2019).

Another limitation of this study was that due to the restrictions of the Covid-19 pandemic this intervention had to be delivered remotely, via teleconferencing. Yet, the sparse evidence on remotely delivered speech and language interventions has demonstrated equitable outcomes to in lexical word retrieval interventions for people with PPA (Dial et al., 2019), and exploration of remote delivery of communication partner training interventions for PPA are emerging (Beeke et al., 2021). Thus, this paper adds to the developing literature demonstrating that speech and language interventions can be delivered to people with dementia remotely, specifically people with PCA. A better understanding of barriers and facilitators to accessing speech and language therapy for people with PCA and other non-PPA dementia’s, may inform implementation and tailoring of interventions within a clinical setting.

## Conclusions

This case study describes how a person-centered communication partner training program, tailored to the needs of people with PPA (BCPPA), was used to support a person living with PCA and their communication partner. Whilst this study does not present empirical evidence that all people living with progressive aphasia associated with PCA or any other rare dementia will make gains, participants subjectively reported benefit and speech and language interventions such as this may hold promise in improving interactions between people with PCA and their communication partners. Future studies should explore further explore the utility of communication partner training for people with PCA and their family members and utilize objective measures to evaluate the perceived benefits reported in our study. Moreover, further research is urgently required to develop a better understanding of the speech and language needs, and in turn drive development of appropriate interventions for people with PCA.
